# Effects of warming and drought on growth and development of soybean in Hailun region

**DOI:** 10.1515/biol-2022-0717

**Published:** 2023-10-03

**Authors:** Qi Li, Dekyi Droma, Xipeng Sun, Yunfa Qiao, Zhenghua Hu, Xuying Zhang

**Affiliations:** School of Applied Meteorology, Nanjing University of Information Science & Technology, Nanjing, China

**Keywords:** WOFOST model, soybean warming, drought, output

## Abstract

As a result of global warming, drought, flooding, change in the rainfall pattern, etc. occur frequently. All these natural disasters could cause serious damage to the food security. Soybean is one of the most important oil crops in China. In recent years, the changing climate has brought many uncertain risks to the growth and production of soybean. In this study, based on the local meteorological, soil, and soybean growth-related experimental data, the effects of high temperature and drought stress on soybean were tested. The test parameters were leaf area index (LAI) and dry matter weight, while the analytical tool used was World Food Studies Model crop model. The research was carried out in Hailun City, Heilongjiang Province, China. The results showed that warming stress shortened the growth period of soybean and reduced the LAI and dry matter accumulation. On the other hand, drought stress also showed a significant impact on the growth period as well as reduced LAI and dry matter accumulation. Comparing the whole growth as well as the flowering-stage to seed-filling-stage treatments of soybean, the results were found very similar. It indicated that the soybean growth from flowering to seed-filling stage was strongly affected by the external environmental factors. The high temperature and drought disasters in the fruiting stages would have a greater impact on the growth and production of soybean crop.

## 1 Introduction

Global warming, mainly due to human activities, is the main cause of many current climate challenges [1,2,3]. As a result of global warming, an increase in drought, flooding, and other extreme natural disasters occur frequently [4], which may cause serious harm to the food security [5]. Continuous warming and frequent occurrences of extreme weather events have posed a serious threat to China’s agricultural development [6]. Soybean (*Glycine max* (L.) Merr., Family: Poaceae) is one of the most important oil crops and also the main agricultural product particularly in the northeast China [7,8]. However, in recent years, the changing climatic conditions have brought many uncertain risks to the growth of soybean, thereby affecting its production.

After years of development, World Food Studies Model (WOFOST), a crop model, has been improved significantly, including large-scale calibration to evaluate crop parameters such as winter crop overwintering and vernalization processes [9]. Cheng et al. [10] used EnKF algorithm to bring remote sensing soil moisture parameter into the WOFOST model. It improved the analysis of correlation between observed and simulated yield and was found more conducive to crop yield prediction at the field scale. Pan et al. [11] have combined the time-series multispectral remote sensing data collected by unmanned aerial vehicle with the WOFOST model. They have used three methods to predict the leaf area index (LAI) of crops at different growth stages accurately and timely. All these have played a significant role in formulating appropriate planting schemes and avoiding disasters. The WOFOST model is also widely used in China [12,13], but there are few studies on soybean growth simulation.

Heilongjiang Province is a major soybean production based in China. The annual average planting area and the total output of soybean account for 30 and 35%, respectively, of the country. The province alone has been contributing to 80% of the total export volume of soybean for China. In recent years, China’s soybean industry has been facing new challenges. So, in the new era, improving quality and increasing self-sufficiency level of the soybean production system have become a key task for the development of soybean industry in China [14]. In this study, the WOFOST model was used to analyze the sensitivity and localization of the model parameters. This was achieved by combining the historical, meteorological, soil, and soybean yield data into the WOFOST model, which verified the parameters to bring the simulation in line with the growth of soybean. In order to explore the crop field response of soybean to future high-temperature or drought stress conditions, different phases of life of soybean such as the whole growth stage and flowering-stage to seed-filling stage were simulated. The simulation was performed by increasing temperature and drought stress. Therefore, the purpose of this study was to help soybean production line to achieve the goal of disaster prevention and/or reduction and stable yield increase, and to provide the basis for the local decision-making departments to adopt appropriate cultivation strategies and management countermeasures.

## 2 Materials and methods

### 2.1 Overview of the study area

The study area is located in the center of the black soil region of Hailun City, Heilongjiang Province (47°39′N, 126°50′E), northeast China. It is a flat terrain experiencing temperate continental monsoon climate, high temperature, rainy summer, and with cold and dry winter. The annual average temperature is 1.5°C, and the annual total rainfall ranges from 500 to 600 mm. This area is suitable for the growth of soybean, where the growth period generally ranges from May to August. In this study, the WOFOST model was verified by the actual soybean yield data collected from the Hailun Experimental Station of Chinese Academy of Sciences for 2004, 2009, 2015, 2017, and 2018 production years. Soybean and maize crop rotation has been conventionally practiced in the study area. The data sets used in the model were from discontinuous production years with varied experimental treatment methods of planting soybean. In this study, the same experimental approach is selected to deal with the soybean yield data.

### 2.2 Data sources

The meteorological and field data, used in this study, were from the natural growth state of soybean [15–17]. The meteorological data required for the WOFOST model operation did include daily minimum and maximum temperature, solar radiation, water vapor pressure, wind speed, and rainfall. However, the data on solar radiation were not directly measured but daily observed sunshine hours were used to calculate the same, using the Angstrom [18] equation. Soil data did include soil types, soil physical and chemical properties, soil bulk density, field capacity and saturation porosity, moisture content, and other physical and chemical properties. Those were mainly acquired from the Chinese soil database (http://vdb3.soil.csdb.cn/). According to the previous research results and soil properties in Heilongjiang Province, the soil parameters are adjusted [19]. Table 1 shows the main soil parameter values of the WOFOST model.

**Table 1:** Main soil parameter values of the WOFOST model

Parameter	Meaning	Unit	Value
SMW	Wilting coefficient	%	20.3
SMFCF	Field moisture capacity	%	31.4
SM0	Saturated moisture	%	38.5

### 2.3 Parameter calibration of the WOFOST model

It is difficult to calibrate each parameter accurately because of the difference of varieties and field management. Therefore, it is necessary to calibrate the sensitivity of the input parameters. The developed concept for calibration was based on: to select the more sensitive parameters to modify and to use the default values of the model or the reference values in the literature directly for the less sensitive parameters. One-at-a-time method is the most common method to calibrate the model parameters, which omits the correlation between the parameters and is convenient to establish the sensitivity of each independent parameter [20]. The WOFOST model is run for simulation, and the result is observed after the parameter value to be determined is adjusted up or down by 10% without changing the simulation environment and other parameter values under the potential growth condition [21].

In this study, TAGP (total aboveground production), TWSO (total dry weight of storage organs), TWST (total dry weight of stems), and TWLV (total dry weight of leaves) of the model output results were selected as comparison items. And the change percentage of each index, namely, the sensitivity of the selected parameters, was calculated, as shown in the following formula:

(1) \[S=\frac{{Y}_{i}-{Y}_{0}}{{Y}_{0}},]\]

where *Y*_0_ is the simulation result of the initial parameter value of the model, and *Y*_i_ is the model simulation result after the change of its parameter.

Select the parameters with higher sensitivity and larger value range, such as TMNFTB and AMAXTB2, and use the trial-and-error method to further determine the appropriate values of the parameters. The specific parameter values are shown in Table 2.

**Table 2:** Main crop parameters of the WOFOST model

Parameter	Meaning	Unit	Value
TDWI	Initial dry matter weight	kg/hm^2^	120
TMUS2	Accumulated temperature from flowering to maturity	°C day	1150
SLATB1	Specific leaf area	hm²/kg	0.014
SPAN	Leaf life cycle at 35°C	day	23
CVL	Efficiency of conversion of assimilate into dry weight of leaf	—	0.72
CVR	Efficiency of assimilation into root dry matter	—	0.72
CVS	Efficiency of assimilation into stem dry matter	—	0.69
Q10	Rate of increase in respiration rate with a temperature change of 10°C	—	2
CVO	Efficiency of conversion of assimilate into storage organ	—	0.48

### 2.4 Select base year

In this study, by comparing the relative deviation of temperature and precipitation anomaly index and yield, a general representative year was selected as the base year to facilitate the simulation of stress treatment in the later period, and the anomaly results are shown in Figure 3. The calculation formula of anomaly index is shown in the following formula:

(2) \[{\mathrm{Anomaly\; index}}=\frac{\hspace{1em}{\mathrm{Average\; temperature\; per\; year}}-{\mathrm{Fifteen}}\text{-}{\mathrm{year\; mean\; temperature}}}{{\mathrm{Fifteen}}\text{-}{\mathrm{year\; mean\; temperature}}}]\]

According to the results, the temperature anomaly index in 2018 was the lowest, reaching −0.035, but the precipitation anomaly value was too high, the precipitation and temperature anomaly values in 2015 were both at a small level, but the relative deviation of yield simulation was high, the temperature anomaly index in 2017 was 0.106, and the precipitation anomaly index was −0.2. The relative deviation of the yield was −0.10, which belonged to a low level, so it was used as the base year (CK) for the follow-up study.

## 3 Results and analysis

### 3.1 Verification of the WOFOST model

3.1

The simulation verification indexes include mean absolute error (MAE), mean relative error (MRE), root mean square error (RMSE), and relative root mean square error (RRMSE). See formulas (3), (4), (5), and (6) for specific calculation, respectively:

(3) \[\text{MAE}=\frac{1}{n}\mathop{\sum }\limits_{i=1}^{n}| {B}_{i}-{G}_{i}| ,]\]

(4) \[\text{MRE}=\frac{1}{n}\mathop{\sum }\limits_{i=1}^{n}\left|\frac{{B}_{i}-{G}_{i}}{{B}_{i}}\right|,]\]

(5) \[\text{RMSE}=\sqrt{\frac{{\sum }_{i=1}^{n}{({B}_{i}-{G}_{i})}^{2}}{n}},]\]

(6) \[\text{RRMSE}=\frac{\text{RMSE}}{\overline{{B}_{i}}}\times 100 \% ,]\]

where *B*_*i*_ and *G*_*i*_ represent the observed value and the simulated value, respectively; *B*_*i*_ is the average value of the observed value; *n* is the number of comparative data; MAE and MRE represent the conformity degree of the simulated value and the observed value, respectively; and RMSE and RRMSE represent the fitting precision of the simulated value and observed value, and the smaller the value is, the better the simulation effect is. The calculation results are shown in Table 3. It can be seen from Table 3 that the RRMSE value of the WOFOST model for soybean yield simulation reached 3.5%, and the simulation effect for yield was better.

**Table 3:** Comparative analysis of simulated and observed yield

Numerical value	Result
Measured mean (kg/hm^2^)	2488.4
Simulated mean (kg/hm^2^)	2,477
MAE (kg/hm^2^)	77
MRE (%)	0.031
RMSE (kg/hm^2^)	86.113
RRMSE (%)	3.5

**Figure 1:**
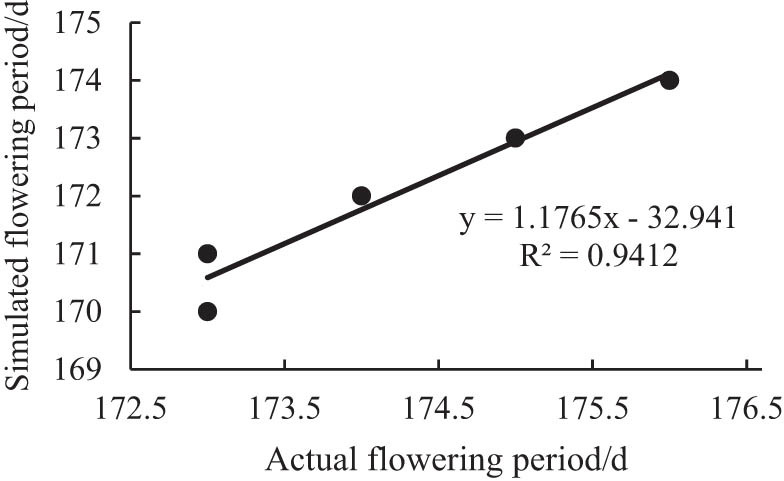
Comparison of simulated and measured flowering date.

**Figure 2:**
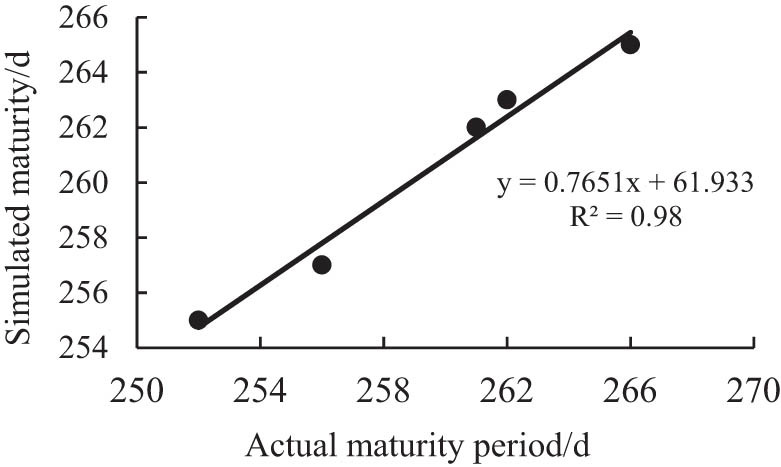
Comparison of simulated and measured values in mature period.

Figures 1 and 2 are the comparisons between the simulated flowering period and maturity period and the actual corresponding growth period. It can be seen from the figure that the *R* ^2^ of the simulated and measured values of the soybean flowering period is 0.94, and the *R* ^2^ of the simulated and measured values of the soybean maturity period is 0.98. The linear fitting precision of both is high, indicating that the simulation effect is good.

The aforementioned results showed that the WOFOST model simulated soybean yield and growth period results are relatively accurate, after adjusting the parameters of the local model in the study area of soybean growth and development of dynamic simulation effect is good, and the adjusted WOFOST model can be used for Hailun area of soybean growth and yield simulation.

### 3.2 Coercive settings

Flowering and podding stages are the most important periods of soybean growth and development wherein the ovary gradually expands to form young pods. This period is also the most efficient water and fertilizer requirement period of soybean. So, adequate water and heat conditions can promote the formation and growth of pods, a prerequisite for seed-filling stage and yield formation. Seed-filling stage is the period of expanding seeds in the pod, which is the most critical period of soybean seed formation. During this period, the common high temperature and drought and flood disasters will lead to pod death, shriveled grain, grain weight decline, and other phenomena, which directly lead to soybean yield reduction. In this study, two groups of experiments were designed: one is the whole growth period of soybean treatment experiment and the other is the only for soybean flowering to seed-filling stage treatment, using the output results of the WOFOST model as an index, to compare the response of soybean to high temperature and drought stress in different stages.

The specific coercion setting method is modified based on the quasi-annual meteorological data. Among them, the setting of warming treatment in the whole growth period was to increase the average, minimum, and maximum temperature of soybean growth period by 1, 2, and 3°C, respectively. The setting of the warming treatment group from the flowering stage to the seed-filling stage is that only the warming treatment is carried out in this stage, and no treatment is carried out in other growth stages, and the setting of the warming stress is the same as that of the treatment group in the whole growth period.

In the WOFOST model, the simulation method under water-limited condition is usually used to simulate the drought situation, which can more truly simulate the physiological state of crops under water-limited condition. The drought stress in this study was mainly regulated by precipitation. The specific drought stress setting is set according to the precipitation anomaly percentage. That is, the precipitation during the simulation period is changed based on the original precipitation anomaly, so that the modified precipitation anomaly percentage-integrated value is maintained at three different levels, and the specific drought standard is shown in Table 4.

**Table 4:** Table of soybean drought grades

Level	Precipitation anomaly percentage (%)
Light drought	70
Moderate drought	60
Severe drought	50

### 3.3 Effects of warming on the LAI of soybean

The LAI is an important index reflecting the growth status of plant population, which can mainly limit the interception and accumulation of light energy by crops, thus affecting the photosynthesis and respiration of crops, and is closely related to the yield of soybean [22]. The dry matter accumulation of soybean ear is the direct embodiment of soybean yield. Therefore, in this study, the LAI and dry matter accumulation of soybean were used as indicators for comparative analysis.

**Figure 3:**
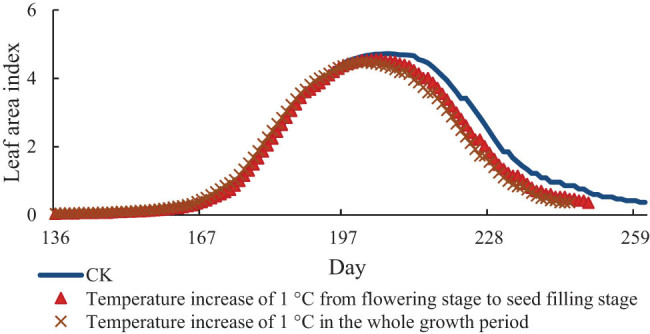
LAI of soybean at 1°C.

**Figure 4:**
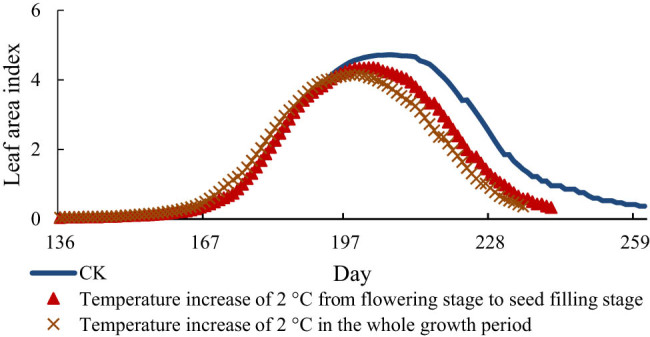
LAI of soybean at 2°C.

**Figure 5:**
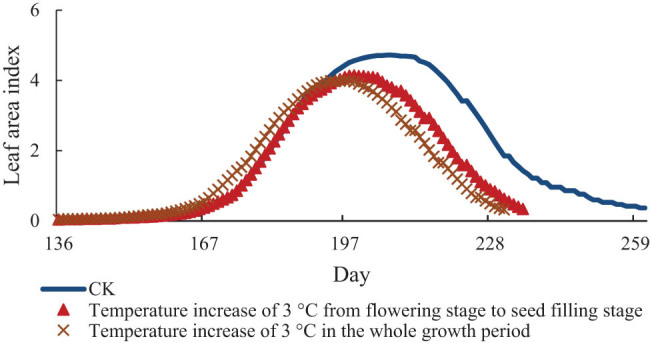
LAI of soybean at 3°C.

Figures 3–5 show the change values of the LAI of soybean under the conditions of increasing temperature of 1, 2, and 3°C, respectively. The LAI of soybean increases continuously from the early flowering stage, reaches the maximum value at the seed-filling stage, and then decreases rapidly. It also decreased with the increase in temperature. The LAI of the treatment group reached the maximum on the 201st day, i.e., 6 days earlier than that under the normal temperature, and the LAI was 4.47. From this value, the LAI decreased by 0.25 compared with that under the normal temperature. Starting from flowering to seed-filling stage, the treatment group reached the maximum on the 204th day, 3 days earlier than the normal temperature, and the LAI decreased by 0.16. When the temperature increased 2°C, the LAI of the treatment group in the whole growth period reached the maximum on the 198th day, which was 9 days earlier than the normal temperature and decreased by 0.57. The LAI of the treatment group from flowering stage to seed-filling stage reached the maximum on the 202nd day, which was 5 days earlier than the normal temperature and decreased by 0.37. Under the condition of 3°C warming, the LAI of the treatment group reached the maximum on the 194th day, 13 days earlier than the actual temperature and the LAI decreased by 0.73. The treatment group reached the maximum on the 199th day from flowering to seed-filling stage, 8 days earlier than the normal temperature and decreased by 0.58.

### 3.4 Effects of warming on dry matter accumulation of soybean

**Figure 6:**
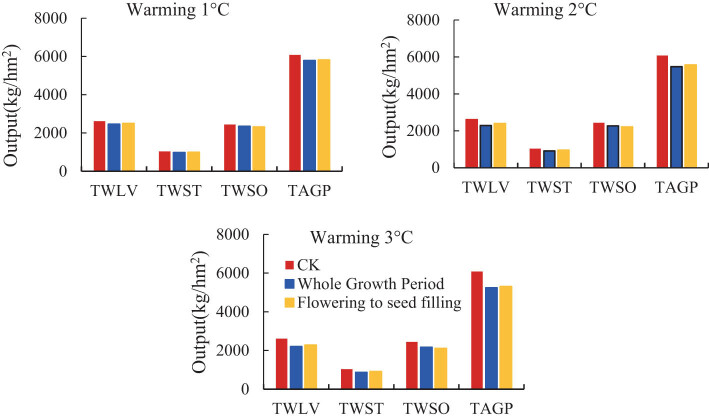
Dry matter accumulation of soybean under warming strip.

Figure 6 shows the dry matter accumulation of soybean under different warming conditions, in which TWLV, TWST, TWSO, and TAGP are the total dry weight of leaves, stems, storage organs, and aboveground production, respectively. The yield per plant is determined by the number of pods per plant, the number of seeds per pod, and the weight of seeds. The available position of soybean is mainly the position of seeds. Therefore, TWSO (ear weight) is mainly used as the basis of yield when discussing the yield. Compared with the three sets of charts, warming affected the dry matter accumulation of each part, except for TWSO yield; the yield of the other three parts was less than that of the treatment group from flowering to seed-filling stage in the whole growth period and less than that of the CK treatment group. The results of TWSO showed that the yield of the treatment group from anthesis to seed-filling stage was less than that of the treatment group from the whole growth period.

### 3.5 Effects of drought on LAI of soybean

**Figure 7:**
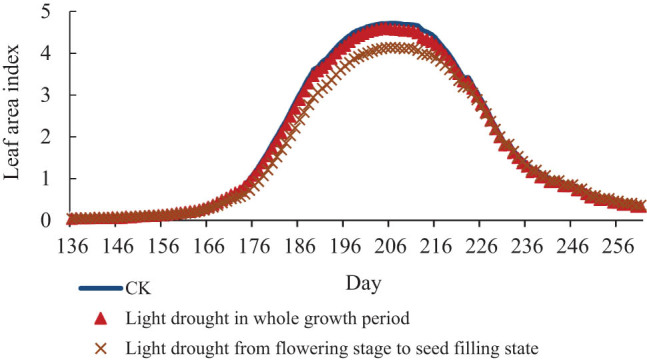
LAI of soybean under light drought stress.

**Figure 8:**
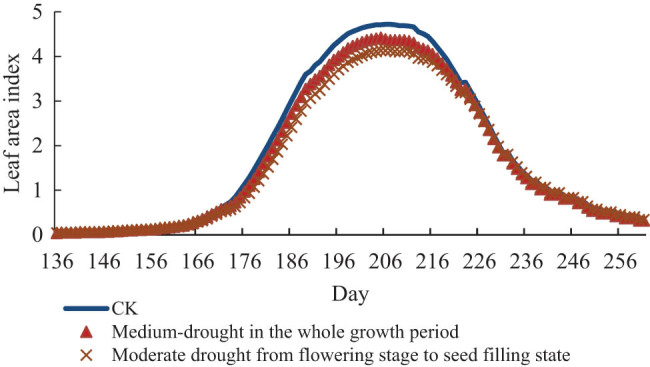
LAI of soybean under moderate drought stress.

**Figure 9:**
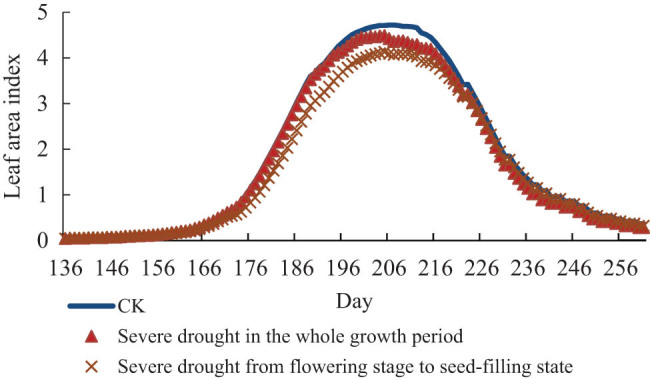
LAI of soybean under severe drought stress.

Figures 7–9 represents the charts of the LAI of soybean under different drought stress. The results show that the three degrees of drought stress all reduce the value of the LAI of soybean in different degrees. But there are no effects on the growth period of soybean, and the time when the LAI reaches the maximum value in the same group of treatments is on the same day. Under light drought stress, the maximum leaf area of treatment group in the whole growth period was 4.6, which was decreased by 0.12 compared with CK, and the maximum leaf area of treatment group from flowering stage to seed-filling stage was 4.14, which was decreased by 0.58 compared with CK. Under moderate drought stress, the maximum leaf area of treatment group in the whole growth period was 4.42, which was 0.30 lower than that of CK, and the maximum leaf area of treatment group from flowering stage to seed-filling stage was 4.13, which was 0.57 lower than that of normal temperature. Under severe drought stress, the maximum leaf area of treatment group in the whole growth period was 4.49, which decreased by 0.23 compared with CK, and the maximum leaf area of treatment group from flowering stage to seed-filling stage was 4.13, which decreased by 0.57 compared with normal temperature.

### 3.6 Effects of drought on dry matter accumulation of soybean

**Figure 10:**
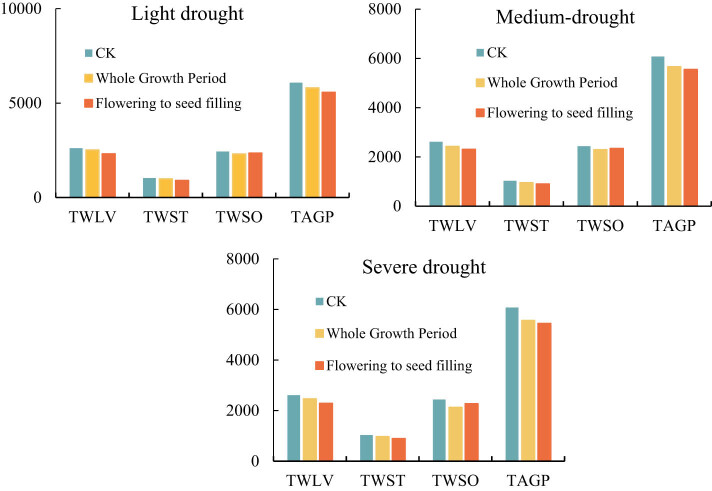
Dry matter accumulation of soybean under different stages of drought stress.

Figure 10 is a dry matter accumulation diagram of soybean under drought stress, and the yield of all parts under drought stress shows that the yield of the treatment group in the whole growth period is less than that of the treatment group in the flowering period to the seed-filling period and less than that of the CK treatment group. The yield reduction rate of TWSO was calculated by comparing the spike weight and yield of each treatment with the control group. Under light drought stress, the yield reduction rate of the treatment group from flowering to seed-filling stage and the whole growth period were 3.5 and 5.5%, respectively, but under the moderate drought stress, the rates were 3.9 and 6.08%, respectively. The reduction rate of the treatment group in the whole growth period was 12.57%. In conclusion, drought reduced the yield of soybean, especially from flowering stage to seed-filling stage.

## 4 Analysis and discussion

Leaf physicochemical characteristics (active leaf area and physiological reactions) are very sensitive to heat and water stresses, which in many cases are interrelated and have an accumulative effect on plant growth [1,3]. In this study, two different growth stages (i.e., whole growth period and flowering to seed-filling stage) of soybean were tested with stress factors. Comparing the two groups of experiments, it was found that both the high temperature and drought stress reduced the LAI of soybean. It is worth mentioning that although the second group of experiments were devoted to only flowering to seed-filling stage, the decline of the LAI was more prominent than that of the whole growth period. This phenomenon and soybean leaf growth rate have a certain relationship, which means flowering stage to seed-filling period is the most vigorous stage of growth in soybean leaves. At this stage, excessive temperature accelerates leaf transpiration, while insufficient water inhibits the growth of plant leaves. The LAI refers to the multiple of the total area of plant leaves per unit land area. Therefore, high temperature and drought stress at this stage will inevitably have a greater impact on the LAI of soybean, and several studies have shown that drought stress significantly reduces the level of crop LAI [23].

From the model of soybean LAI simulation results, the change of the LAI and the degree of drought were significantly negatively correlated. It indicates that low soil moisture will also affect the photosynthesis and respiration of soybean, leading to the reduction of soybean leaf area, limiting the accumulation of photosynthates, and ultimately causing plant yield reduction. Therefore, water is an important factor for soybean production; more clearly, adequate soil moisture can improve the growth of soybean and increase the number of soybean pods [24]. Increasing the LAI, especially at seed-filling stage, was beneficial to the accumulation of photosynthate and grain yield [25,26].

Comparing the dry matter accumulation of soybean spike in the two groups, the difference between the warming and drought stress groups from flowering to seed-filling stage and the whole growth period treatment group was very small. The reason of this phenomenon is that the net photosynthetic rate (Pn) is constantly changing in the whole growth period of soybean. It indicates, roughly, that the Pn increases slowly from the flower bud differentiation stage, increases rapidly at the flowering stage, reaches the highest value at the pod-setting stage, then decreases slowly at the pod-filling stage, and decreases to the lowest value at the maturity stage. Therefore, the main dry matter accumulation period of soybean via photosynthesis occurs during the flowering to the pod-setting stage. Previous studies have also shown that high temperature and drought stress affect the photosynthetic capacity of soybean leaves, which is not conducive to flower opening and fertilization process, but reduce the podding rate and affect dry matter accumulation, thereby reducing the yield of soybean [27,28,29]. This finding is consistent with the conclusion of this study.

## 5 Conclusion

In this study, the localization of the WOFOST model was completed by using the actual data of Hailun City, Heilongjiang Province, and the effects of warming and drought on soybean growth and development in different periods were simulated using the same model. The results showed that the growth period of soybean was significantly shortened, and the LAI and dry matter accumulation were reduced in different degrees by warming treatment in the whole growth period of soybean. The results of warming and drought treatments were different. The growth period of soybean was not shortened, but the LAI and dry matter accumulation were significantly reduced. The yield of each part of soybean was further simulated, which showed a significant decrease after drought stress. The yield reduction rate increased with the deepening of drought but showed a reduced rate in case of stem and leaf dry weight and was significantly higher than that of ear weight.

In the experiment of increasing temperature and drought stress treatment from flowering to seed-filling stage of soybean, it was found that the stress simulation results of this stage were very close to those of the treatment groups in the whole growth stage. The reduction of the LAI under drought treatment was stronger than that of the whole growth period treatment but the yield of TWSO was slightly less than it. All the other simulation results showed that the effect of stress treatment on the whole growth period was slightly greater than that on the stress treatment only from flowering to seed-filling stage. This indicated that the growth of soybean was greatly affected by the external environment from the flowering to the seed-filling stage, and the high temperature or drought stress at this stage would seriously damage the growth and development of soybean.
